# The complete chloroplast genome sequences of *Lychnis wilfordii* and *Silene capitata* and comparative analyses with other Caryophyllaceae genomes

**DOI:** 10.1371/journal.pone.0172924

**Published:** 2017-02-27

**Authors:** Jong-Soo Kang, Byoung Yoon Lee, Myounghai Kwak

**Affiliations:** Plant Resources Division, National Institute of Biological Resources, Incheon, Republic of Korea; National Renewable Energy Laboratory, UNITED STATES

## Abstract

The complete chloroplast genomes of *Lychnis wilfordii* and *Silene capitata* were determined and compared with ten previously reported Caryophyllaceae chloroplast genomes. The chloroplast genome sequences of *L*. *wilfordii* and *S*. *capitata* contain 152,320 bp and 150,224 bp, respectively. The gene contents and orders among 12 Caryophyllaceae species are consistent, but several microstructural changes have occurred. Expansion of the inverted repeat (IR) regions at the large single copy (LSC)/IRb and small single copy (SSC)/IR boundaries led to partial or entire gene duplications. Additionally, rearrangements of the LSC region were caused by gene inversions and/or transpositions. The 18 kb inversions, which occurred three times in different lineages of tribe *Sileneae*, were thought to be facilitated by the intermolecular duplicated sequences. Sequence analyses of the *L*. *wilfordii* and *S*. *capitata* genomes revealed 39 and 43 repeats, respectively, including forward, palindromic, and reverse repeats. In addition, a total of 67 and 56 simple sequence repeats were discovered in the *L*. *wilfordii* and *S*. *capitata* chloroplast genomes, respectively. Finally, we constructed phylogenetic trees of the 12 Caryophyllaceae species and two Amaranthaceae species based on 73 protein-coding genes using both maximum parsimony and likelihood methods.

## Introduction

Chloroplasts are important photosynthetic organelles that provide energy for the synthesis of glucose, fatty acids, and amino acids [1,2]. The chloroplast genome is the smallest of the plant genomes, ranging from 135 to 160 kb in most plants [3–5]. Most angiosperm chloroplast genomes have a quadripartite circular structure and contain two copies of inverted repeat (IR) regions, separating a large single copy (LSC) region and small single copy (SSC) region [5]. Recently, with the rapid development of next-generation sequencing platforms, many chloroplast genome sequences have been reported and used to help resolve plant phylogenies [6,7]. Chloroplast genomic data are widely used in various studies, such as those on molecular phylogeny, molecular identification (DNA barcoding), and genetic diversity [8–10]. The structure and gene order of the chloroplast genome are stable, and the rates of nucleotide substitution are generally slow in angiosperms [11–14].

Rearrangements in the chloroplast genome were considered to have occurred rarely enough in evolution that they can be used to demarcate major groups [4]; however, recently some lineages have revealed various patterns of changes in chloroplast genomes, for example large-scale rearrangements, gene duplications, and even loss of IR regions [15–20]. Scattered angiosperm lineages show extensive rearrangement of plastid genomes, and these gene order changes are correlated with increased rates of nucleotide substitutions and gene and intron losses [6]. Rearrangements of the chloroplast genome are often associated with repeated sequences [5].

The family Caryophyllaceae consists of 75–80 genera and approximately 2,000 species, which are widely distributed, mainly in the temperate or warm-temperate regions of the northern hemisphere [21]. The genera *Lychnis* and *Silene* are sister genera belonging to tribe *Sileneae*, but the taxonomic identities and limitations between these two genera remain unclear [21–23], which is why the genus *Lychnis* was nested within *Silene* using nuclear ribosomal internal transcribed spacer (nrITS), five chloroplast genes, and intergenic spacers (IGS) [24]. Previous studies have shown that the *Sileneae* underwent accelerated plastid genome evolution, including inversions, shifts in IR boundaries, large indels, intron losses, and rapid rates of amino acid sequence substitution [25,26]. Interestingly, the *psaA*-*ycf3*:*psaI*-*ycf4* inversion and intron losses in *clpP*-1 and *clpP*-2 were suggested to be independent events that occurred three times [26].

A total of ten Caryophyllaceae chloroplast genomes have been reported [25–28]. In the genus *Lychnis*, only the chloroplast genome of *L*. *chalcedonica* has been reported [26], whereas in *Silene*, chloroplast genomes from a total of six species have been reported [25,26]. Therefore, in this study, we sequenced the complete chloroplast genomes of *L*. *wilfordii* and *S*. *capitata* and then analyzed them to identify their genetic characteristics and differences compared with other Caryophyllaceae species. The specific goals of the present study were to (1) present the complete chloroplast genome sequences of two *Sileneae* species, (2) investigate any significant characteristics suggesting extensive genome rearrangement in this tribe, and (3) explore significant changes in gene content and intron losses in the tribe *Sileneae*.

## Materials and methods

### Plant materials, DNA extraction, sequencing and genome assembly

Leaf materials from *Lychnis wilfordii* and *Silene capitata* were obtained from living plants by seed germination in a greenhouse at the Korean Botanical Garden. The voucher specimens of *L*. *wilfordii* (NIBRVP0000542331) and *S*. *capitata* (NIBRVP0000542433) were deposited in the National Institute of Biological Resources Herbarium (KB). Total genomic DNA was extracted using the Genome Wizard kit (Promega, Madison, WI, USA). Sequencing libraries were prepared using the NEXTflex Rapid DNA-seq kit (Bioo Scientific, Austin, TX, USA). Paired-end sequencing libraries containing insert sizes of approximately 350–450 bp were sequenced on the Illumina Hiseq 2500 platform (Illumina Inc., San Diego, CA, USA) at the National Instrumentation Center for Environmental Management (Seoul, South Korea), yielding 27,739,600 reads from *L*. *wilfordii* and 22,127,152 reads from *S*. *capitata*, each with a read length of 250 bp. These paired-end reads were aligned with sequences from *Silene vulgaris* (JF715057). After screening these paired-end reads through alignment with *S*. *vulgaris* plastid genome, 585,206 (2.1%) reads of *L*. *wilfordii* and 661,807 (2.9%) reads of *S*. *capitata* were extracted with mean of coverage 980× and 1082×, respectively. *De novo* assembly was performed using Geneious v. 7.1.3 (Biomatters, Auckland, New Zealand). The consensus sequences were extracted and gap-filled by PCR amplification using specific primers based on the gaps between sequences. The PCR products were purified and sequenced by Sanger sequencing.

### Genome annotation and comparative analyses

The initial annotation of the two Caryophyllaceae chloroplast genomes was performed using Dual Organellar GenoMe Annotator (DOGMA) [29]. From this initial annotation, putative starts, stops, and intron positions were determined by comparison with homologous genes in other Caryophyllaceae chloroplast genomes. The tRNA genes were annotated using DOGMA and tRNAscan-SE [30]. The circular chloroplast genome map was drawn using the OGDraw program [31]. The complete chloroplast genomes of *L*. *wilfordii* and *S*. *capitata* were compared with those of ten other Caryophyllaceae species using the mVISTA program in Shuffle-LAGAN mode (Table 1) [32]. *Agrostemma githago* (KF527884) was used as a reference.

**Table 1:** GenBank accession numbers and references used in this study.

Family name	Scientific name	Accession numbers	Reference
Caryophyllaceae	*Agrostemma githago*	KF527884	Sloan et al., 2014
	*Colobanthus quitensis*	KT737383	Kang et al., 2015
	*Dianthus longicalyx*	KM668208	Gurusamy et al., 2016
	*Lychnis chalcedonica*	KF527886	Sloan et al., 2014
	*Lychnis wilfordii*	KT727929	In this study
	*Silene capitata*	KT727930	In this study
	*Silene conica*	JF715054	Sloan et al., 2012
	*Silene conoidea*	KF527885	Sloan et al., 2014
	*Silene latifolia*	JF715055	Sloan et al., 2012
	*Silene noctiflora*	JF715056	Sloan et al., 2012
	*Silene paradoxa*	KF527887	Sloan et al., 2014
	*Silene vulgaris*	JF715057	Sloan et al., 2012
Amaranthaceae	*Beta vulgaris*	KR230391	Stadermann et al., 2015
	*Salicornia europaea*	KJ629116	Unpublished

### Repeat sequence analysis

Simple sequence repeats (SSRs or microsatellites; mono-, di-, tri-, tetra-, penta-, and hexanucleotide repeats) were detected using Phobos v. 3.3.12 [33] with thresholds of ten repeat units for mononucleotide SSRs, five repeat units for di- and trinucleotide SSRs, and three repeat units for tetra-, penta-, and hexanucleotide SSRs. REPuter [34] was also used to analyze the repeat sequences, which included forward, reverse, palindromic, and complementary sequences with a minimal length of 30 bp and 90% sequence identities (Hamming distance of three). Moreover, we constructed a phylogenetic trees based on the sequences of the pairs of repeat regions to investigate the relationship between the distributions of repeat sequences and structural inversions. Maximum parsimony (MP) analysis was conducted using PAUP v. 4.0a150 [35], and branch support was assessed using 1000 bootstrap replicates.

### Phylogenetic analysis

Phylogenetic analyses based on 73 protein-coding genes were also performed for 12 Caryophyllaceae species, using two Amaranthaceae species (*Beta vulgaris* and *Salicornia europaea*) as the outgroup (Table 1). Among 77 whole protein-coding genes, *ycf1*, *ycf2*, *accD*, *clpP* genes were excluded from data matrix, since those genes were reported fast evolving genes with high substitution rate within tribe *Sileneae* [26]. Consequently, a total of 54,271 bp were aligned using MAFFT [36]. MP analysis was conducted using PAUP v. 4.0a150 [35], and branch support was assessed using 1000 bootstrap replicates. Before Maximum likelihood (ML) analysis, a search for the best fitting substitution model was performed using jModeltest v. 2.1.5 [37]. Based on the Akaike Information Criterion (AIC) and Akaike Information Criterion with Correction (AICc), GTR+I+G was the best model. ML analysis was performed using RAxML v. 7.4.2 with 1000 bootstrap replicates and the GTR+I+G model [38]. Bayesian inference was performed using MrBayes 3.2 [39].

## Results and discussion

### Genome organization and features

**Fig 1:**
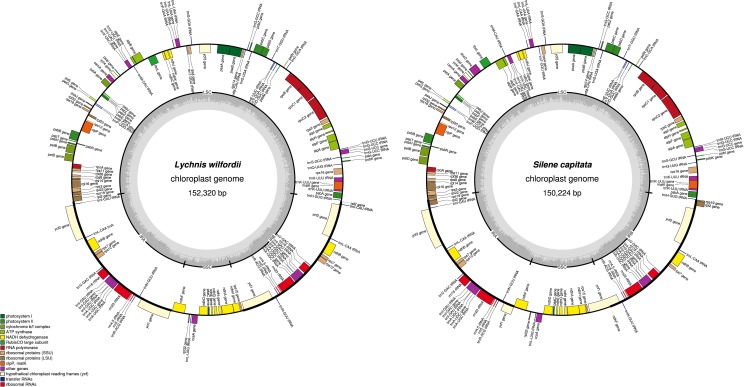
The chloroplast genomes of *Lychnis wilfordii* and *Silene capitata*. Genes inside the circle are transcribed clockwise, while genes outside are transcribed counter-clockwise. The dark gray inner circle corresponds to the GC content and the light-gray circle to the AT content.

The complete sizes of the *L*. *wilfordii* and *S*. *capitata* chloroplast genomes are 152,320 and 150,224 bp, respectively (Fig 1, Table 2). The size of the *L*. *wilfordii* chloroplast genome is the longest among the reported Caryophyllaceae species. The *L*. *wilfordii* and *S*. *capitata* genomes include a pair of IRs of 27,709 bp and 25,371 bp separated by a SSC region of 12,914 bp and 17,313 bp and a LSC region of 83,988 bp and 82,169 bp, respectively (Fig 1, Table 2), similar to the published Caryophyllaceae chloroplast genomes [25–28]. The *L*. *wilfordii* chloroplast genome contains 110 unique genes, 17 of which are duplicated in the IR region, giving a total of 127 genes (Fig 1, Table 2, S1 Table). The *S*. *capitata* chloroplast genome contains 111 unique genes, 19 of which are duplicated in the IR region, giving a total of 130 genes (Fig 1, Table 2, S2 Table). The chloroplast genomes of these two species contain 30 distinct tRNAs, seven of which are duplicated in the IR region. Seventeen genes contain one or two introns: 14 contain one intron and three (*rps12*, *clpP*, and *ycf3*) two introns. Six of the genes containing one intron are tRNAs (S1 and S2 Tables).

**Table 2:** Summary of chloroplast genome characteristics of two caryophyllaceae genomes.

Genome features	*Lychnis wilfordii*	*Silene capitata*
Size (bp)	152,320	150,224
LSC length (bp)	83,988	82,169
SSC length (bp)	12,914	17,313
IR length (bp)	27,709	25,371
Number of genes	110	111
Protein-coding genes	76+6	77+8
tRNA genes	30+7	30+7
rRNA genes	4+4	4+4
Number of genes duplicated in IR	17	19
Overall GC content (%)	36.6	36.3
GC content in LSC (%)	34.9	34.2
GC content in SSC (%)	31.2	29.8
GC content in IR (%)	40.5	42.2

In addition, while the *L*. *wilfordii* and *S*. *capitata* chloroplast genomes both have lost the *infA* gene, the *accD* gene was pseudogenized only in *L*. *wilfordii*. The lack or pseudogenization of the *infA* gene has been discovered in many taxa outside of Caryophyllaceae, such as the Brassicaceae, Fabaceae, Liliaceae, Malvaceae, and Onagraceae [25,26,40–44]. Loss or pseudogenization of the *accD* gene in the plastid genome or *accD* gene transfer to the nucleus has also been reported in various angiosperm lineages, including Poaceae, Orobanchaceae, Ericaceae, and Primulaceae [45–48].

### Comparative chloroplast genomic analysis

**Fig 2:**
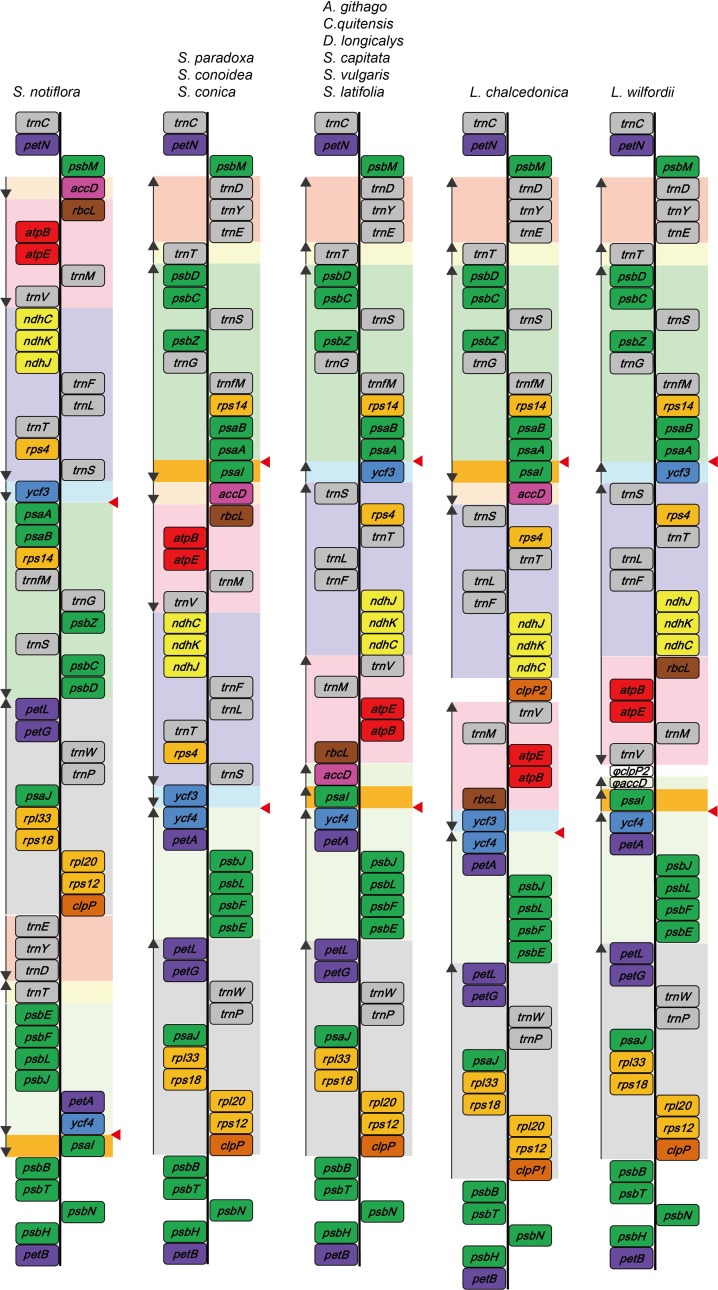
Comparison of gene rearrangements in the large single copy region among 12 Caryophyllaceae. Genes are indicated in the colored boxes. Green: photosystem; blue: hypothetical chloroplast reading frame (*ycf* series); yellow: NADH-dehydrogenase; light orange: ribosomal subunit; dark orange: protease; brown: rubisco subunit; red: ATP synthase; purple: cytochrome b/f complex; pink: acetyl-CoA carboxylase; gray: tRNA; white: pseudogene. The larger boxes indicate that the inversion or transposition fragments have been identified. The arrows to the left of the large boxes indicate the direction of inversion compared with the ancestral large single copy gene order of this region. The red triangle to the right of the large squares indicates the breaking point of an 18 kb inversion with intermolecular duplicated sequences.

We compared gene arrangements in the chloroplast genomes of *L*. *wilfordii* and *S*. *capitata* with those of the ten previously reported Caryophyllaceae species (Fig 2). The chloroplast genome of *S*. *capitata* has an identical gene order with those of the genera *Agrostemma*, *Colobanthus*, and *Dianthus*, but the chloroplast genome of *L*. *wilfordii* has unique structural changes compared with previously reported Caryophyllaceae chloroplast genomes (Fig 2). The gene rearrangements present in the LSC regions were a result of inversions and/or transpositions (Fig 2). The chloroplast genome of *L*. *wilfordii* revealed an inversion of the *trnV-rbcL* region compared with the genomes of other genera (Fig 2), whereas the *L*. *chalcedonica* genome had twice the number of inversions and transpositions in the *accD-psaI* and *ycf3* regions compared with the genomes of other genera (Fig 2). Interestingly, truncated partial sequences of *clpP*-2 and *accD* were found in the IGS region between *trnV* and *psaI*. The 5’ upstream non-genic region and a partial 348 bp sequence of the *accD* gene, as well as the exon 1 and partial intron 1 sequences of *clpP*-2, have remained, but the downstream regions of both genes were truncated in the *L*. *wilfordii* chloroplast genome. Compared with the gene orders in other chloroplast genomes, these disruptions in the *accD* and *clpP*-2 genes may have occurred by inversion of the *trnV-rbcL* fragment. Thus, we deduced that duplication of *clpP* occurred before diversification of *L*. *chalcedonica* from *L*. *wilfordii*, and that transposition of *psaI-accD* and the loss of introns in *clpP*-1 and *clpP*-2 in *L*. *chalcedonica* may have occurred after species diversification.

In the genus *Silene*, we identified three types of chloroplast genomes (Fig 2). These are a) the common type of chloroplast genome observed in most Caryophyllaceae (*Agrostemma*, *Colobanthus*, and *Dianthus*) (seen in *S*. *capitata*, *S*. *latifolia*, and *S*. *vulgaris*); b) chloroplast genomes exhibiting an inversion of the *ycf3-psaI* regions (seen in *S*. *paradoxa*, *S*. *conoidea*, and *S*. *conica*); c) chloroplast genomes exhibiting transpositions and/or inversions of the *psbD-accD*, *petL-clpP*, *trnD-T*, and *psaI-psbE* regions (seen in *S*. *noctiflora*). *Silene noctiflora* currently has the most complicated chloroplast genome among the Caryophyllaceae.

**Fig 3:**
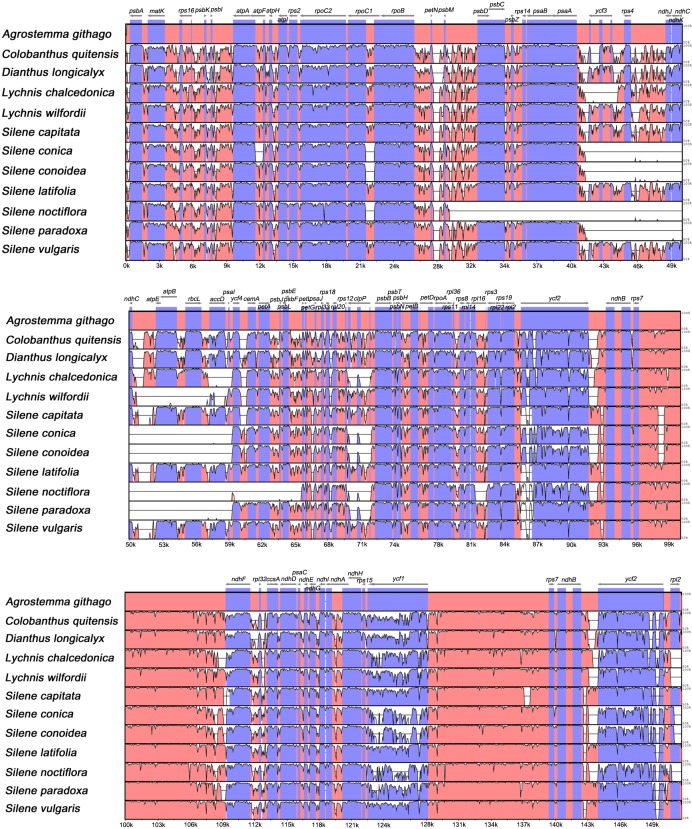
Sequence alignment of 12 Caryophyllaceae genomes in mVISTA, using the *Agrostemma githago* genome as a reference. The vertical scale indicates the identity percentage, ranging from 50 to 100%.

Overall sequence identity was analyzed with mVISTA program the among the 12 chloroplast genomes of Caryophyllaceae, using the *Agrostemma githago* genome as a reference (Fig 3). The results revealed higher divergence in the LSC regions than in the IRs and SSCs, as a result of gene rearrangements (Figs 2 and 3), and greater conservation in the coding regions than in the non-coding regions (Fig 3). The most divergent coding regions were the *ycf1*, *ycf2*, *accD*, and *clpP* genes, which are similar to results from previous studies [25,26,49], showing lower (under 50%) similarity compared with other protein-coding regions (Fig 3). Consequently, we suggest that these genes evolve rapidly in Caryophyllaceae (including the tribe *Sileneae*). These genes are either absent or highly variable in the genomes of Campanulaceae, Geraniaceae, and Poaceae [6].

### Boundaries between single copy and inverted repeat regions

**Fig 4:**
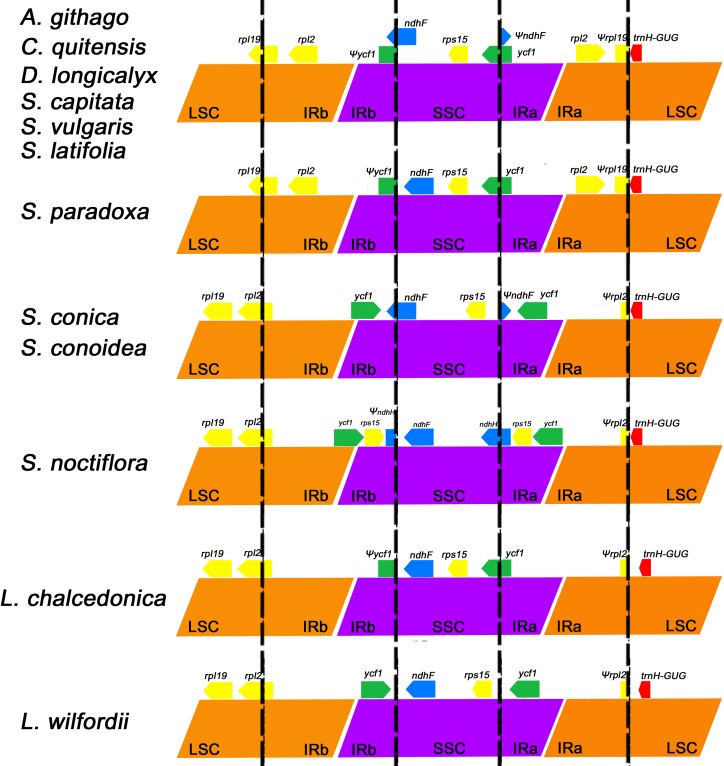
Comparison of the large single copy, inverted repeat, and small single copy border regions among 12 Caryophyllaceae chloroplast genomes.

The size variations among angiosperm chloroplast genomes are mostly the result of expansion or contraction of the IR region [50]. Additionally, the expansion or contraction of the IR region differs among various plant species [51]. In this study, the LSC-IR and IR-SSC boundaries of the 12 sequenced Caryophyllaceae genomes were compared (Fig 4). IR locations have changed substantially in *Lychnis* and *Silene* as a result of movement of the boundaries between the IR and SC regions (Fig 4). The IR and SC boundaries of *S*. *capitata* are consistent with those of the *S*. *vulgaris* and *S*. *latifolia* genomes, as well as the Caryophyllaceae genera *Agrostemma*, *Colobanthus*, and *Dianthus* (Fig 4).

The expansion of the IR at the SSC/IR boundary that duplicates the entire *ycf1* gene was found only in the genome of *L*. *wilfordii* and three *Silene* species (*S*. *conica*, *S*. *conoidea*, and *S*. *noctiflora*). This event was observed in non-core Caryophyllales [52]. In the *S*. *noctiflora* chloroplast genome, the *ycf1* and *rps15* genes are duplicated within the IR region (Fig 4), and this species contains the longest IR region (29,891 bp) among the 12 Caryophyllaceae species. However, the contraction of the IR at the LSC/IR boundary that duplicates a part of the *rpl2* gene was found only in *Silene* (*S*. *conica*, *S*. *conoidea*, and *S*. *noctiflora*) and *Lychnis* (*L*. *wilfordii* and *L*. *chalcedonica*). *Lychnis chalcedonica* has the shortest IR region (23,540 bp) among 12 Caryophyllaceae species due to contraction of the IR region at the LSC/IR boundary and lack of expansion of the IR region at the IR/SSC boundary.

### Repeat sequence analysis and short inverted repeats as inversion hotspots

**Fig 5:**
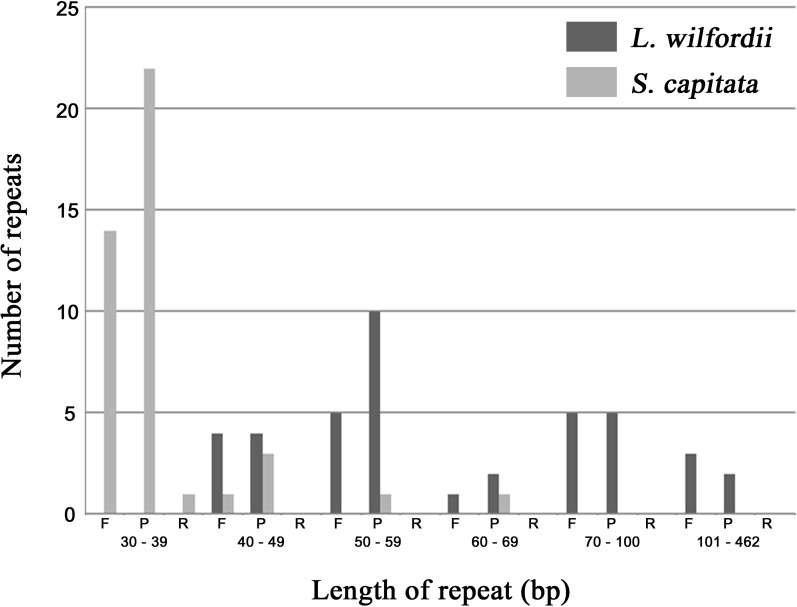
Frequency of repeat sequences in the chloroplast genomes of *Lychnis wilfordii* and *Silene capitata* using REPuter.

We analyzed repeat sequences from the chloroplast genomes of *L*. *wilfordii* and *S*. *capitata* and observed forward, reverse, palindromic, and complementary repeats using REPuter. *Lychnis wilfordii* contains 17 forward repeats and 22 palindromic repeats, whose lengths range from 40 to 462 bp (Fig 5, S3 Table). *Silene capitata* contains 15 forward repeats, 27 palindromic repeats, and only one reverse repeat, whose lengths range from 30 to 64 bp (Fig 5, S4 Table). Most of the *L*. *wilfordii* repeats are located in IGS regions (56.4%), and less than half were located in genes (30.8%; *ycf1* and *ycf2*) and introns (12.8%; *ndhA* and *clpP* intron). In contrast, the majority of *S*. *capitata* repeats are located in genes (44.0%; *ycf2*, *ycf4*, *psaA*, *psaB*, *ccsA*, *trnS-GGA*, *trnS-GCU*, *trnS-UGA*, *trnG-UCC*, and *trnG-GCC*), with fewer located in IGSs (40.0%) and introns (16.0%; *ycf3*, *rpl16*, *rpoC1*, and *ndhA* introns).

We then analyzed the SSRs (or microsatellites), which are increasingly evaluated in molecular genetic studies because of their high reproducibility, ease of scoring, and fast throughput compared with other marker techniques [53]. In the *L*. *wilfordii* and *S*. *capitata* chloroplast genomes, the most abundant SSRs were A or T mononucleotide repeats, which accounted for approximately 77.6% and 76.8% of the total SSRs, followed by tetranucleotides (10.4% and 16.1%) and dinucleotides (10.4% and 7.1%), respectively (Table 3, S5 and S6 Tables). SSRs in the chloroplast genome are commonly composed of A or T repeats and rarely G or C repeats [54,55]. Furthermore, the majority of *L*. *wilfordii* and *S*. *capitata* SSRs are located in IGS regions (49.3% and 55.4%), followed by genes (37.3% and 26.8%) and introns (13.4% and 17.9%), respectively (S5 and S6 Tables). SSRs located in coding regions were found mainly in *ycf1* and *rpoC2*, with the remaining SSRs found in *matK*, *rpoA*, *psbF*, *atpB*, and *atpF*. Among the SSRs in genes, part or all of those in *matK*, *rpoC2*, *rpoA*, *psbF*, *ycf1*, and *rrn23* were shared by the two Caryophyllaceae species.

**Table 3:** The types and number of SSRs in chloroplast genomes of *L*. *wilfordii* and *S*. *capitata*.

	*L*. *wilfordii*	*S*. *capitata*
Mononucleotide repeats	52	43
Dinucleotide repeats	7	4
Trinucleotide repeats	1	0
Tetranucleotide repeats	7	9
Total SSRs in cp genome	67	56
Total SSRs in protein-coding genes	25	14

Penta- and hexanucleotides were not discovered over than three repeats.

**Fig 6:**
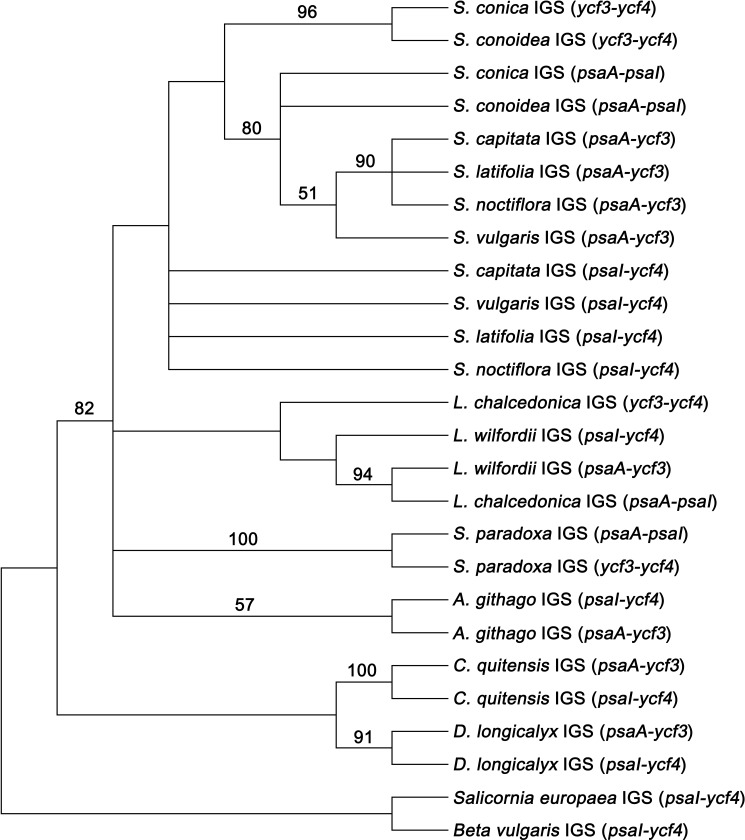
A phylogenetic tree of the inverted repeats at the end of the 18 kb inversion of the *psaI*-*ycf3* fragment. Two intermolecular repeat sequences on both sides of the inversion were extracted. For two Amaranthaceae species (*Salicornia europaea* and *Beta vulgaris*), only one homologous sequence was present. Gene names in parentheses indicate the intergenic location between the two genes where both repeat sequences are present.

Under the assumption that the common chloroplast types observed in most Caryophyllaceae (*Agrostemma*, *Colobanthus*, and *Dianthus*, *S*. *capitata*, *S*. *latifolia*, and *S*. *vulgaris*) are ancestral, the inversion of the *ycf3*-*psaI* fragment might have occurred independently at least three times: in *L*. *chalcedonica*, *S*. *notiflora*, and the lineage containing *S*. *conoidea* and *S*. *conica*, consistent with previous results [26]. Interestingly, loss of introns in the *clpP* gene is always coupled with these inversions. In Caryophyllaceae, all 12 species possess imperfect palindromic repeats on both sides of the *ycf3*-*psaI* fragment (Fig 2), whereas only one homologous sequence corresponding to these repeats was found in the intergenic region between *psaI* and *ycf4* in two Amaranthaceae species. In all cases, the repeat sequences were overlapped by partial *ycf4* coding region sequences (63 bp). Thus, the partial *ycf4* and upstream sequences might have been duplicated in the IGS between *psaA* and *ycf3* before diversification of Caryophyllaceae. Even in the repeats between *psaA* and *ycf3*, expected to be non-genic sequences, intermolecular duplicated sequences were grouped together in *A*. *githago*, *C*. *quitensis*, *D*. *longicalix*, and *S*. *paradoxa* based on the maximum parsimony tree (Fig 6). The intermolecular duplicated sequences were not grouped together in the other *Silene* species. A large fragment inversion mediated by short IRs was reported in several plant species. The 22 kb inversion in Asteraceae [56], the 42 kb inversion in *Abies* of the Pinaceae [57], the 21 kb inversion in *Jasminae* of the Oleaceae [16], and the 36 kb inversion in the core Genistoids are thought to be induced by IRs in tRNAs or repeat elements several base pairs long. These dispersed repeats were shown to promote inversions via intermolecular recombination [5,58,59]. Thus, we suggest that this short IR in Caryophyllaceae might mediate intramolecular flip-flop recombination events, and thus, independent identical inversion events of the *ycf3*-*psaI* 18 kb fragment might be facilitated independently in different lineages.

### Phylogenetic analysis

**Fig 7:**
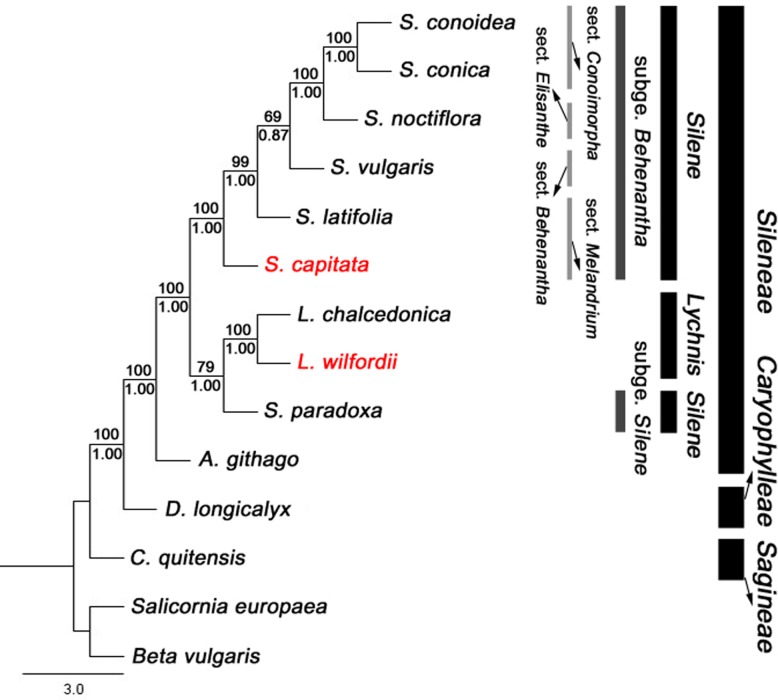
Phylogenetic tree of 14 taxa based on 73 protein-coding genes using the maximum likelihood method. Taxa in red are the new genomes reported in this study. Bootstrap values greater than 50% are shown above the nodes, and the Bayesian posterior probabilities are shown below the nodes.

Both the MP and ML trees of the 12 Caryophyllaceae species and two Amaranthaceae species based on 73 protein-coding genes showed consistent phylogenetic patterns (Fig 7, S1 Fig). In the ML tree, bootstrap analysis indicated that eight of ten nodes were supported by bootstrap values ≥ 99% and the other two nodes by values > 65%. In previous studies, the genus *Lychnis* was shown to be nested within the genus *Silene* based on internal transcribed spacer (ITS) sequences of nuclear genome and chloroplast DNA data [24,60] and based on chloroplast genome data [26]. *Lychnis* species were nested within *Silene*, close to *S*. *paradoxa* in the subgenus *Silene*, which is consistent with previous studies [26] (Fig 7). The subgenus *Behenantha* and monophyly of sect. *Melandrium* (*S*. *capitata* and *S*. *latifolia*) were not supported, whereas *S*. *conoidea* and *S*. *conica* of sect. *Conoimorpha* form a monophyletic group were found to be closely related to *S*. *noctiflora* of sect. *Elisanthe* (Fig 7). However, we need additional chloroplast genome data from more *Sileneae* species to resolve the relationship between *Lychnis* and *Silene*, as well as the infrageneric relationships of *Silene*.

## Supporting information

S1 Fig Phylogenetic tree of 14 taxa based on 73 protein-coding genes obtained using the maximum parsimony (MP) method in PAUP. Bootstrap values greater than 50% are shown above the nodes.
(TIF)

S1 Table List of genes present in the chloroplast genome of *Lychnis wilfordii*. (DOCX)

S2 Table List of genes present in the chloroplast genome of *Silene capitata*. (DOCX)

S3 Table List of repeat sequences in the chloroplast genome of *Lychnis wilfordii*. (DOCX)

S4 Table List of repeat sequences in the chloroplast genome of *Silene capitata*. (DOCX)

S5 Table List of simple sequence repeats in the chloroplast genome of *Lychnis wilfordii*. (DOCX)

S6 Table List of simple sequence repeats in the chloroplast genome of *Silene capitata*. (DOCX)
