# Supplementary figures and images for: The complete chloroplast genome sequences of Lychnis wilfordii and Silene capitata and comparative analyses with other Caryophyllaceae genomes
Source: PLoS One. 2017 Feb 27;12(2):e0172924. doi: 10.1371/journal.pone.0172924 (PMC5328339; doi:10.1371/journal.pone.0172924)

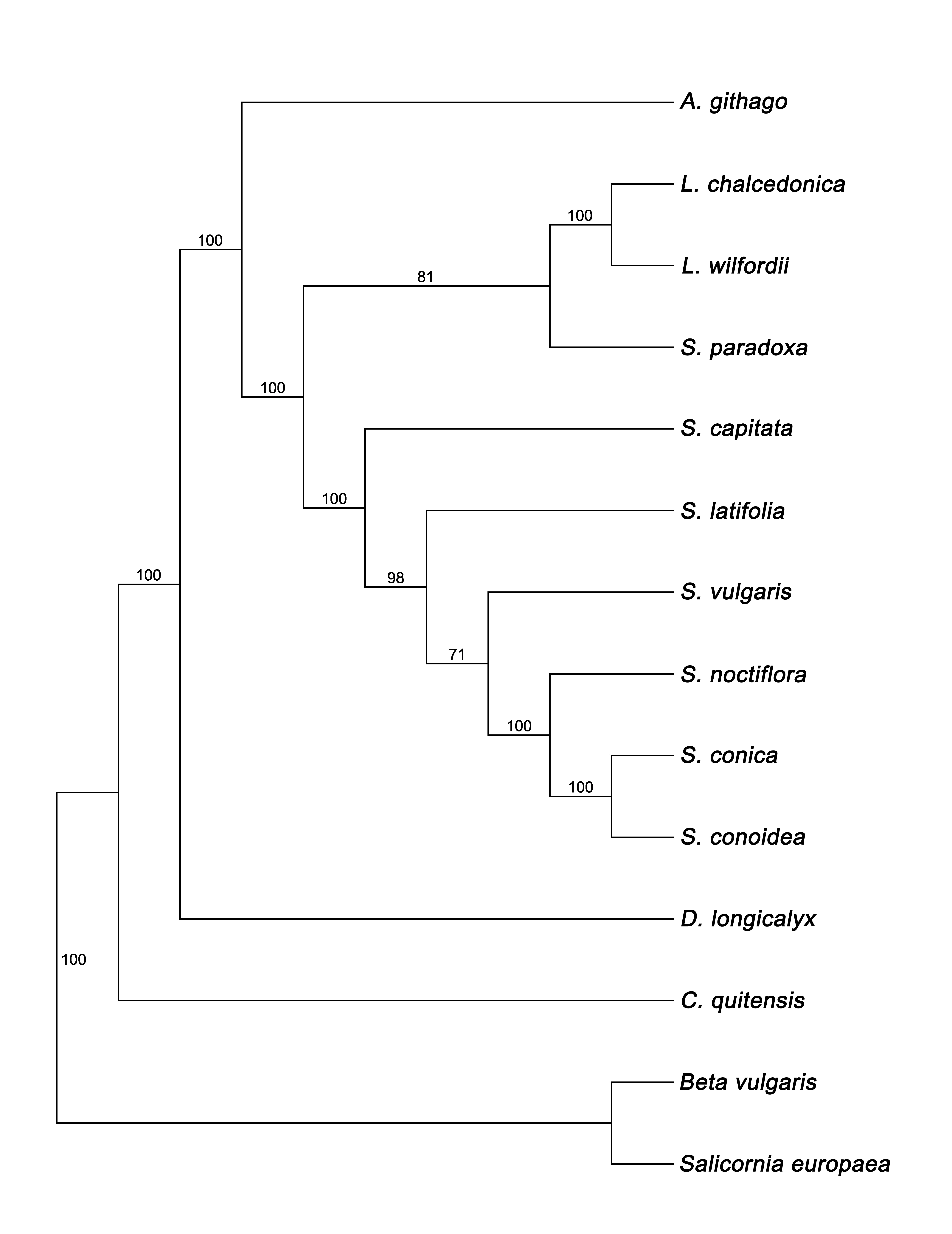

Supplement: S1 Fig — Bootstrap values greater than 50% are shown above the nodes. (TIF) [file pone.0172924.s001.tif]
