# Supplementary material for: The complete chloroplast genome sequences of Lychnis wilfordii and Silene capitata and comparative analyses with other Caryophyllaceae genomes
Source: PLoS One. 2017 Feb 27;12(2):e0172924. doi: 10.1371/journal.pone.0172924 (PMC5328339; doi:10.1371/journal.pone.0172924)
Supplement: S1 Table — (DOCX) [file pone.0172924.s002.docx]

S1 Table. List of genes present in the chloroplast genome of *Lychnis wilfordii*.

| DNA function | Group of genes | Name of genes |
| --- | --- | --- |
| Self replication | Ribosomal RNAs | *rrn16* (×2), *rrn23* (×2), *rrn4.5* (×2), *rrn5* (×2) |
|  | Transfer RNAs | *trnA-UGC** (×2)*, trnC-GCA, trnD-GUC, trnE-UUC, trnF-GAA, trnfM-CAU, trnG-GCC, trnG-UCC*, trnH-GUG, trnI-CAU* (×2)*, trnI-GAU** (×2)*, trnK-UUU*, trnL-CAA* (×2)*, trnL-UAA*, trnL-UAG, trnM-CAU, trnN-GUU* (×2)*, trnP-UGG, trnQ-UUG, trnR-ACG* (×2)*, trnR-UCU, trnS-GCU, trnS-GGA, trnS-UGA, trnT-GGU, trnT-UGU, trnV-GAC* (×2)*, trnV-UAC*, trnW-CCA, trnY-GUA* |
|  | Small subunit of ribosome | *rps2, rps3, rps4, rps7* (×2)*, rps8, rps11, rps12** (×2)*, rps14, rps15, rps16*, rps18, rps19* |
|  | Large subunit of ribosome | *rpl2* (×2, part)*, rpl14, rpl16*, rpl20, rpl22, rpl32, rpl33, rpl36* |
|  | DNA dependent RNA polymerase | *rpoA*, *rpoB, rpoC1*, rpoC2* |
| Genes for  photosysthesis | Subunit of NADH-dehydrogenase | *ndhA*, ndhB** (×2)*, ndhC, ndhD, ndhE, ndhF, ndhG, ndhH, ndhI, ndhJ, ndhK* |
|  | Subunit of photosystem Ⅰ | *psaA, psaB, psaC, psaI, psaJ* |
|  | Subunit of photosystem Ⅱ | *psbA, psbB, psbC, psbD, psbE, psbF, psbH, psbI, psbJ, psbK, psbL, psbM, psbN, psbT, psbZ* |
|  | Subunit of cytochrome b/f complex | *petA, petB*, petD*, petG, petL, petN* |
|  | Subunit of ATP synthase | *atpA, atpB, atpE, atpF*, atpH, atpI* |
|  | Large subunit of rubisco | *rbcL* |
| Potential protein-coding genes | Maturase | *matK* |
|  | Translational initiation factor | Ψ*infA* |
|  | Protease | *clpP*** |
|  | Envelop membrane protein | *cemA* |
|  | Subunit of Acetyl-CoA carboxylase | Ψ*a*ccD |
|  | c-type cytochrome synthesis gene | *ccsA* |
| Genes of  unknown function | Conserved open reading frames | *ycf1* (×2)*, ycf2* (×2)*, ycf3**, ycf4* |

*, genes in intron; **, genes in two introns; ×2, duplicated genes; Ψ, pseudogene
