# Supplementary material for: The complete chloroplast genome sequences of Lychnis wilfordii and Silene capitata and comparative analyses with other Caryophyllaceae genomes
Source: PLoS One. 2017 Feb 27;12(2):e0172924. doi: 10.1371/journal.pone.0172924 (PMC5328339; doi:10.1371/journal.pone.0172924)
Supplement: S3 Table — (DOCX) [file pone.0172924.s004.docx]

S3 Table. List of repeat sequences in the chloroplast genome of *Lychnis wilfordii*.

| **Repeat Size** | **Type** | **Start of 1^st^ repeat** | **Start the repeat found in other region** | **Location** | **Region** |
| --- | --- | --- | --- | --- | --- |
| 40 | F, P | 106679 | 106739, 129529 | ycf1 | IR |
| 41 | F | 68 | 102 | IGS (trnH-GUG-rpl2) | LSC |
| 42 | F, P | 95338 | 121441, 140928 | IGS (trnV-GAC-rps12), ndhA intron | IR |
| 46 | P | 46356 | 46356 | IGS (trnL-UAA-trnT-UGU) | LSC |
| 50 | P | 118182 | 118182 | IGS (psaC-ndhD) | SSC |
| 51 | P | 43911 | 58051 | IGS (trnS-GGA-ycf3), IGS(trnV-UAC-psaI) | LSC |
| 52 | P | 26667 | 26667 | IGS (trnC-GCA-rpoB) | LSC |
| 52 | P | 51143 | 56917 | IGS (rbcL-ndhC), IGS (trnV-UAC-psaI) | LSC |
| 54 | F, P | 105764 | 105803, 130451, 130490 | IGS (trnN-GUU-ycf1) | IR |
| 56 | P | 58623 | 58623 | IGS (trnV-UAC-psaI) | LSC |
| 56 | P | 66437 | 66437 | IGS (rpl33-psaJ) | LSC |
| 57 | F, P | 106689 | 106719, 129532, 129562 | ycf1 | IR |
| 59 | F | 59 | 4743 | IGS (trnH-GUG-rpl2 (part)), IGS (trnK-UUU-rps16) | LSC |
| 61 | P | 40964 | 58041 | IGS (psaA-ycf3), IGS(trnV-UAC-psaI) | LSC |
| 64 | F | 56075 | 56140 | IGS (trnM-CAU-trnV-UAC) | LSC |
| 74 | P | 40835 | 59110 | IGS (psaA-ycf3), IGS(psaI-ycf4) | LSC |
| 74 | P | 57623 | 70471 | IGS (trnV-UAC-psaI), clpP intron1 | LSC |
| 82 | F, P | 90131 | 90155, 146071, 146095 | ycf2 | IR |
| 86 | F | 57882 | 57945 | IGS (trnV-UAC-psaI) | LSC |
| 94 | P | 40722 | 59193 | IGS (psaA-ycf3), IGS(psaI-ycf4) | LSC |
| 100 | F | 4804 | 43640 | IGS (trnK-UUU-rps16), IGS (trnS-GGA-ycf3) | LSC |
| 152 | P | 57511 | 70505 | IGS (trnV-UAC-psaI), clpP intron1 | LSC |
| 400 | F | 40901 | 43848 | IGS (psaA-ycf3), IGS(trnS-GGA-ycf3) | LSC |
| 403 | P | 57074 | 70740 | IGS (trnV-UAC-psaI), clpP intron1 | LSC |
| 415 | F | 40980 | 43927 | IGS (psaA-ycf3), IGS(trnS-GGA-ycf3) | LSC |
| 462 | F | 40909 | 43856 | IGS (psaA-ycf3), IGS(trnS-GGA-ycf3) | LSC |
