# Supplementary material for: The complete chloroplast genome sequences of Lychnis wilfordii and Silene capitata and comparative analyses with other Caryophyllaceae genomes
Source: PLoS One. 2017 Feb 27;12(2):e0172924. doi: 10.1371/journal.pone.0172924 (PMC5328339; doi:10.1371/journal.pone.0172924)
Supplement: S4 Table — (DOCX) [file pone.0172924.s005.docx]

S4 Table. List of repeat sequences in the chloroplast genome of *Silene capitata*.

| **Repeat Size** | **Type** | **Start of 1^st^ repeat** | **Start the repeat found in other region** | **Location** | **Region** |
| --- | --- | --- | --- | --- | --- |
| 30 | R | 6121 | 6121 | IGS (trnQ-UUG-rps16) | LSC |
| 30 | F, P | 7830 | 43973, 33714 | trnS-GCU, trnS-GGA, trnS-UGA | LSC |
| 30 | F | 21645 | 110242 | rpoC1 intron, IGS (rpl32-ndhF) | LSC, SSC |
| 30 | F | 36889 | 39113 | psaB, psaA | LSC |
| 30 | P | 40565 | 58588 | IGS (psaA-ycf3), ycf4 | LSC |
| 30 | F | 41684 | 41710 | ycf3 intron2 | LSC |
| 30 | F, P | 88693 | 88735, 143628, 143670 | ycf2 | IR |
| 31 | P | 7730 | 7747 | IGS (trnS-GCU-psbI) | LSC |
| 31 | F | 9116 | 34624 | trnG-UCC, trnG-GCC | LSC |
| 31 | P | 40641 | 58494 | IGS (psaA-ycf3), IGS (psaI-ycf4) | LSC |
| 31 | P | 40676 | 58459 | IGS (psaA-ycf3), IGS (psaI-ycf4) | LSC |
| 31 | P | 41383 | 41386 | ycf3 intron2 | LSC |
| 31 | P | 47589 | 47589 | IGS (trnF-GAA-ndhJ) | LSC |
| 31 | F, P | 88689 | 88707, 143655, 143673 | ycf2 | IR |
| 32 | P | 58013 | 58013 | IGS (accD-psbI) | LSC |
| 32 | F, P | 79811 | 136552, 95809 | rpl16 intron1, IGS (trnV-GAC-rps12) | LSC, IR |
| 32 | F, P | 88677 | 88713, 143648, 143684 | ycf2 | IR |
| 32 | F, P | 88711 | 88735, 143626, 143650 | ycf2 | IR |
| 36 | P | 40735 | 40735 | IGS (psaA-ycf3) | LSC |
| 36 | P | 43741 | 43741 | IGS (trnS-GGA-ycf3) | LSC |
| 36 | P | 118039 | 118039 | ndhA intron | SSC |
| 39 | F, P | 42305 | 95800, 136554 | ycf3 intron1, IGS (trnV-GAC-rps12) | LSC, IR |
| 40 | P | 65471 | 65471 | IGS (rpl33-psaJ) | LSC |
| 42 | F, P | 95798 | 117778, 136553 | ccsA, IGS (trnV-GAC-rps12), ndhA intron | SSC, IR |
| 46 | P | 40626 | 58494 | IGS (psaA-ycf3), IGS (psaI-ycf4) | LSC |
| 50 | P | 114431 | 114431 | IGS (trnL-UAG-rpl32) | SSC |
| 64 | P | 13468 | 13468 | IGS (atpH-I) | LSC |
