# Supplementary material for: The complete chloroplast genome sequences of Lychnis wilfordii and Silene capitata and comparative analyses with other Caryophyllaceae genomes
Source: PLoS One. 2017 Feb 27;12(2):e0172924. doi: 10.1371/journal.pone.0172924 (PMC5328339; doi:10.1371/journal.pone.0172924)
Supplement: S5 Table — (DOCX) [file pone.0172924.s006.docx]

S5 Table. List of simple sequence repeats in the chloroplast genome of *Lychnis wilfordii*.

| **Repeat Unit** | **Length (bp)** | **Number of SSRs**  **(Gene/Intron/IGS)** | **Position** |
| --- | --- | --- | --- |
| A | 10 | 13 (6/1/6) | Gene: atpB, psbF, ycf1; Intron: ndhA intron; IGS: rpl20-rps12, rpl22-rps19, trnH-GUG-psbA, trnL-UAA-trnT-UGU, trnL-UAG-rpl32, trnS-GCU-psbI, |
|  | 11 | 5 (3/0/2) | Gene: ycf1; IGS: atpH-I, trnK-UUU-rps16 |
|  | 12 | 3 (2/0/1) | Gene: ycf1; IGS: trnC-GCA-petN |
|  | 13 | 1 (0/0/1) | IGS: rpl16-rps3 |
|  | 15 | 2 (1/0/1) | Gene: matK; IGS: ndhF-rpl32 |
|  | 18 | 2 (0/0/2) | IGS: psbK-I, ycf1-ndhF |
| T | 10 | 11 (4/1/6) | Gene: ycf1; Intron: trnG-UCC intron; IGS: psbM-petN, rbcL-ndhC, trnL-UAA-trnF-GAA, trnP-UGG, psaJ, trnS-UGA-psbC, trnT-GGU-psbD |
|  | 11 | 7 (4/0/3) | Gene: rpoA, ycf1; IGS: petG-L, trnM-UAC-atpE, trnV-UAC-psaI |
|  | 12 | 3 (2/1/0) | Gene: ycf1; Intron: petB intron |
|  | 13 | 1 (1/0/0) | Gene: rpoC2 |
|  | 16 | 1 (0/1/0) | Intron: trnK-UUU intron1 |
|  | 18 | 1 (0/0/1) | IGS: rps15-ycf1 |
| G | 10 | 1 (0/1/0) | Intron: trnK-UUU intron1 |
|  | 11 | 1 (0/0/1) | IGS: psaJ-rpl33 |
| AT | 10 | 2 (1/0/1) | Gene: atpF; IGS: trnS-GCU-psbI |
|  | 14 | 1 (0/0/1) | IGS: trnT-GGU-psbD |
| TA | 10 | 3 (0/2/1) | Intron: trnL-UUU intron; IGS: trnE-UUC-trnT-GGU |
|  | 12 | 1 (0/0/1) | IGS: trnR-UCU-atpA |
| AAT | 18 | 1 (0/1/0) | Intron: trnL-UAA intron |
| ATAA | 12 | 1 (0/0/1) | IGS: rpl33-rps18 |
| AGAA | 12 | 1 (0/0/1) | IGS: psbM-petN |
| AGGT | 12 | 1 (1/0/0) | Gene: rrn23 |
| TTTA | 12 | 1 (0/0/1) | IGS: rps18-rpl20 |
| TTTC | 12 | 2 (0/1/1) | Intron: ycf3 intron2; IGS: psbH-petB |
| CCAT | 12 | 1 (0/0/1) | IGS: trnS-UGA-psbZ |
