# Supplementary material for: The complete chloroplast genome sequences of Lychnis wilfordii and Silene capitata and comparative analyses with other Caryophyllaceae genomes
Source: PLoS One. 2017 Feb 27;12(2):e0172924. doi: 10.1371/journal.pone.0172924 (PMC5328339; doi:10.1371/journal.pone.0172924)
Supplement: S6 Table — (DOCX) [file pone.0172924.s007.docx]

S6 Table. List of simple sequence repeats in the chloroplast genome of *Silene capitata*.

| **Repeat Unit** | **Length (bp)** | **Number of SSRs**  **(Gene/Intron/IGS)** | **Position** |
| --- | --- | --- | --- |
| A | 10 | 9 (2/2/5) | Gene: matK, psbF; Intron: ycf3 intron1, clpP intron1; IGS: psbK-I, trnC-GCA-petN, psaI-ycf4, psbN-H, ndhE-psaC |
|  | 11 | 4 (0/1/3) | Intron: clpP intron2; IGS: trnH-GUG-psbA, atpH-I, trnG-GCC-psbZ |
|  | 14 | 2 (1/0/1) | Gene: rpoC2; IGS: trnT-UGU-rps4 |
|  | 15 | 1 (0/0/1) | IGS: petL-psbE |
|  | 16 | 2 (1/0/1) | Gene: matK; IGS: trnL-UAA-trnT-UGU |
| T | 10 | 14 (6/1/7) | Gene: rpoA, ycf1; Intron: ndhA intron; IGS: trnH-GUG-psbA, rpoC2-rps2, trnV-UAC-ndhC, atpB-rbcL, psbE-petL, petD-rpoA, rpl36-rps11 |
|  | 11 | 5 (2/1/2) | Gene: rpoC2, ycf1; Intron: trnK-UUU intron; IGS: trnM-CAU-atpE, atpB-rbcL |
|  | 12 | 2 (1/1/0) | Gene: rpoC2; Intron: petB intron |
|  | 13 | 2 (0/2/0) | Intron: rpoC1 intron, trnL-UUU intron |
|  | 14 | 2 (0/0/2) | IGS: ndhJ-K, ndhG-I |
| AT | 10 | 3 (0/1/2) | Intron: ycf3 intron2; IGS: trnE-UUC-trnT-GGU, trnL-UAA-trnF-GAA |
| TA | 12 | 1 (0/1/0) | Intron: rpl16 intron |
| AATT | 12 | 1 (0/0/1) | IGS: rpoC2-rps2 |
| AGGT | 12 | 1 (1/0/0) | Gene: rrn23 |
| TAAA | 12 | 1 (0/0/1) | IGS: ycf3-psaA |
| TTTA | 12 | 1 (0/0/1) | IGS: rps18-rpl20 |
| TTTC | 12 | 2 (0/0/2) | IGS: petN-psbM, psbH-petB |
| CCAT | 12 | 1 (0/0/1) | IGS: trnS-UGA-psbZ |
| GAAG | 12 | 1 (0/0/1) | IGS: atpF-H |
| TAAT | 16 | 1 (0/0/1) | IGS: accD-psaI |
